# Effects of soft robotic exosuit on ambulation ability in stroke patients: a systematic review

**DOI:** 10.1186/s12938-023-01150-7

**Published:** 2023-09-05

**Authors:** Ya-Chi Chuang, Yu-Lin Tsai, Tony Tung-Liang Lin, Liang-Jun Ou-Yang, Yu-Chun Lee, Yuan-Yang Cheng, Chuan-Ching Liu, Chun-Sheng Hsu

**Affiliations:** 1 Department of Physical Medicine and Rehabilitation, Taichung Veterans General Hospital, No. 1650 Taiwan Boulevard Sect. 4, Taichung, 407219 Taiwan, ROC; 2 Department of Physical Medicine and Rehabilitation, Chang Gung Memorial Hospital, Linkou, Taoyuan 333423 Taiwan, ROC; 3 Department of Exercise Health Science, National Taiwan University of Sport, Taichung, 404401 Taiwan, ROC; 4 Department of Industrial Engineering and Enterprise Information, Tunghai University, Taichung, 407224 Taiwan, ROC; 5 Department of Post-Baccalaureate Medicine, College of Medicine, National Chung Hsing University, Taichung, 402202 Taiwan; 6 School of Medicine, National Yang Ming Chiao Tung University, Taipei, 112202 Taiwan

**Keywords:** Soft robotic exosuit, Gait, Ambulation, Stroke, Post-stroke hemiparesis

## Abstract

**Background:**

Robot-assisted gait training is incorporated into guidelines for stroke rehabilitation. It is a promising tool combined with conventional therapy for low ambulatory patients. The heavy weight and bulky appearance of a robotic exoskeleton limits its practicality. On the other hand, soft robotic exosuit (SRE) based on its light weight and inconspicuous property, is better tolerated by patients in daily life. The aim of this study is to review the efficacy of the SRE with regard to walking ability and biomechanical properties in stroke patients.

**Methods:**

Electronic searches were carried out in PubMed, Embase, Cochrane Library, Web of Science, and the Physiotherapy Evidence Database. Clinical trials that investigated the effectiveness of SREs on ambulation ability in patients with post-stroke hemiparesis were eligible. Qualitative data synthesis was subsequently performed.

**Results:**

Nine studies were identified as relevant, involving a total of 83 patients. For the assessment of SRE efficacy, outcome measures were walking ability and biomechanical properties. In terms of both immediate effect and training effect, SREs improved the walking speed, walking distance, peak ankle dorsiflexion angle during swing phase, peak paretic propulsion, stride length and compensated gait in stroke patients.

**Conclusions:**

SRE improved the ambulation ability of stroke patients in terms of walking ability and biomechanical properties. The small number of studies limits the generalizability of interpretation. More controlled studies with better quality are required to reach a more solid conclusion on this issue.

**Supplementary Information:**

The online version contains supplementary material available at 10.1186/s12938-023-01150-7.

## Introduction

Normal bipedal locomotion of humans is an elaborate coordinated process composed of adequate joint movements, trunk control, and motions. Diseases such as stroke can impair one’s normal neuromuscular control, resulting in a hemiplegic gait, which is characterized by unilateral hip hiking, leg circumduction, knee hyperextension during stance phase, inadequate propulsion force during late stance phase, drop foot with excessive ankle plantar flexion and further cause reduced foot clearance, decreased walking speed, prolonged double support time, asymmetry joint kinematics and step length [1]. These biomechanical changes consume more energy for the patients, thus posing adverse impacts on ambulation ability [2]. Patients with post-stroke hemiparesis cannot walk easily, resulting in lower daily activities and poorer quality of life [3].

Several methods can be used to help correct post-stroke gait deviation, including traditional gait pattern training, functional electrical stimulation, partial body-weight-supported treadmill training, and robotic assisted training. These training methods when used alone, or combined with others, can improve ambulation ability [4]. Among these methods, robotic assisted training has been recently in the spotlight.

Devices for robotic assisted training are divided into two groups: the rigid exoskeleton and the soft robotic exosuit (SRE). The rigid exoskeleton is a stand-alone equipment or integrated into a body-weight-supported system with excellent support of the hip, knee and ankle joints to those patients with insufficient strength to walk [5–8]. Current stroke rehabilitation guidelines consider exoskeletal wearable robotic devices as a promising way to improve motor function and mobility after stroke when combined with conventional therapy [4]. However, the device is heavy and difficult to don and doff.

An alternative device, the SRE requires less effort for maintaining standing position or during ambulation and is used more often for those chronic stroke patients who have already achieved partial recovery. Typically, SREs are made of functional textile garments that attach to the body at the waist and paretic calf, including actuation module, sensing module and Bowden cables or elastic materials as the medium for force transmission on the body. The actuator types are either electric motors or pneumatic actuators. The sensing module used force or pressure, inertial measurement units or gyroscopes as input [9]. Though SREs provide less support than the rigid exoskeleton, they are more inconspicuous, lightweight, and highly portable in providing mechanical assistance. Due to their less bulky appearance, they serve better as orthotic devices in daily life. Unlike rigid exoskeletons, the effects of SREs on stroke patients have not been fully assessed nor reviewed comprehensively in those patients experiencing stroke and ambulatory dysfunction [7, 10, 11].

Better knowledge of the SRE is indispensable before any further recommendations can be made regarding its use for post-stroke patients. The goal of this review is to determine the efficacy of the SRE, and the ways it affects the biomechanical, functional, and clinical outcomes of patients. In addition, the immediate and post-training effects have been further explored separately. The question we aim to answer through this systematic review is: “Do stroke survivors benefit from the training which involves SREs with regards to walking ability and biomechanical properties?”.

## Results

### Flow of studies through the review

**Fig. 1:**
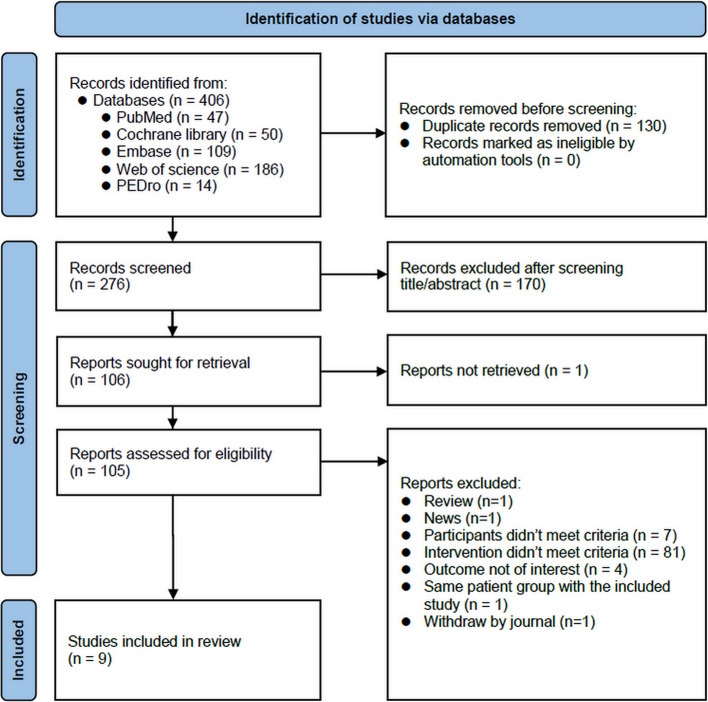
Flow chart according to the statement of PRISMA 2020

The last search was conducted on January 16, 2023, when the authors found 406 studies in their initial search of the 5 databases, with 130 duplications being removed. After screening titles and abstracts, another 170 studies were excluded. The remaining 106 reports were then assessed for eligibility. After reviewing the full manuscripts, those not meeting our inclusion criteria were excluded. In the end, only 9 articles remained and finally entered the review and synthesis. The process is presented in Fig. 1.

### Study characteristics

Of these final 9 reports, 3 were case reports with each one having studied only one participant [12–14], 3 were non-comparative interventional studies [15–17], and 3 were exploratory studies[18–20]. Seven studies were conducted in America and performed by their related research groups, while one study was performed in Russia and one in Korea. The characteristics of these studies are summarized in Table 1.

**Table 1:** Demographic characteristic and training protocol of the included studies

Study	Design	Location	Participants	Type of device	Protocol	Outcome measures (Method)	Follow-up period
Awad et al. (2020) [15]	Non-comparative interventional study	America	N = 36^a^ Mean age = 57 yrs Stroke latency = 7.3 yrs	ReWalk ReStore™ soft robotic exosuit^b^ (Net weight: 5 kg)	5 training sessions in 1 month (20 min on treadmill and 20 min on ground)	Maximal walking speed (10MWT)	About 1 month
Shin et al (2022) [16]	Non-comparative interventional study	America	N = 5 Mean age = 46 yrs Stroke latency = 2.7 yrs	ReWalk ReStore™ soft robotic exosuit^b^ (Net weight: 5 kg)	18 training sessions in 2 months (30 min on treadmill)	Walking speed (10MWT), walking distance (6MWT and 2MWT), Fugl-Meyer scale	About 2 months
Poydasheva et al. (2016) [17]	Non-comparative interventional study	Russia	N = 14 Median age = 53 yrs Stroke latency = 1.2 yrs	Regent soft exoskeleton complex	10 training sessions in 2 weeks + Standard stroke rehabilitation	Walking time (10MWT), Fugl-Meyer scale	About 2 weeks
Awad et al (2017) [19]	Exploratory research	America	N = 9 Mean age = 49 yrs Stroke latency = 4.4 yrs	Soft robotic exosuit (Net weight: 0.9 kg)	Single-session	Maximal walking speed (10MWT), peak dorsiflexion angle^c^, peak paretic propulsion, energy cost	Only measure immediate effect
Sloot et al (2022) [20]	Exploratory research	America	N = 8 Mean age = NS Stroke latency = NS	Soft robotic exosuit^b^ (Net weight: 2 kg)	Single-session	Walking speed (5-min walking test), peak dorsiflexion angle ^c^, peak paretic propulsion, energy cost	Only measure immediate effect
Awad et al (2017) [18]	Exploratory research	America	N = 8 Mean age = 48 yrs Stroke latency = 3.2 yrs	Soft robotic exosuit (Net weight: 0.9 kg)	Single-session	Peak dorsiflexion angle ^c^, hip hiking, hip circumduction, stride length	Only measure immediate effect
Porciuncula et al. (2021) [13]	Case report	America	N = 1 Patient’s age = 54 yrs Stroke latency = 5.6 yrs	Soft robotic exosuit^d^	Robotic Exosuit Augmented Locomotion gait training (REAL gait training): 5 training sessions in 5 consecutive days (30 min on treadmill)	Maximal/comfortable walking test (10MWT), walking distance (6MWT), hip hiking, hip circumduction, stride length	About 1 week
Porciuncula et al. (2019) [12]	Case report	America	N = 1 Patient’s age = 58 yrs Stroke latency = 4.5 yrs	Soft robotic exosuit	6 training sessions(over 2 weeks)	Walking speed (10MWT), walking distance (6MWT), stride length	About 2 weeks
Kwon et al (2019) [14]	Case report	Korea	N = 1 Patient’s age = 49 yrs Stroke latency = 10 months	Soft wearable robotic ankle–foot-orthosis^e^ (Net weight: 1.54 kg) Plantarflexion 30–70 N Dorsiflexion 50 N	Single-session	Walking speed (9-m walking test), walking distance (6MWT), stride length	Only measure immediate effect

N = number, yrs = years, Exp = experimental group, kg = kilogram, 10MWT = 10-m walking test, 6MWT = 6-min walking test, 2MWT = 2-min walking test, N/S = not stated

^a^ Forty-four participants completed safety and reliability evaluation, but only 36 participants completed all training sessions

^b^ Machine provided 25% of the user’s body weight for plantarflexion during push-off and the minimum needed for adequate ground clearance

^c^ Peak ankle dorsiflexion angle during swing phase

^d^ The maximum force was set at 180 N, with onset time at 38–40% of the gait cycle

^e^ Machine provided 30–70 N for plantarflexion and 50 N for dorsiflexion

#### Participants

The studies evaluated 83 patients using SREs, with mean ages ranging from 46 to 58 years of age. All patients experience chronic post-stroke hemiparesis, with a mean stroke latency of 10 months to 7.3 years.

#### Intervention

Net weights of the SREs ranged from 0.9 to 5 kg, with ReWalk ReStore™ being the most frequently used equipment (in ~ 50% of participants). Of note, one of the included studies used soft wearable robotic ankle–foot orthosis [14], with a design similar to other SREs.

#### Comparator

Four studies focused on the immediate effect of the SREs, that is, only a single training session was performed [14, 18–20]. In these research studies, two compared a powered exosuit with an unpowered exosuit, with all of the participants wearing an exosuit [18, 19]. One research study compared those who wore the exosuit with those who did not [20], while the other study made comparisons between those wearing a powered exosuit, those wearing an unpowered exosuit and those not wearing exosuit at all. However, we only documented the results of those wearing a powered exosuit and those not wearing any exosuit [14].

The other five studies focused on the training effect of SREs. All of them compared the data after training with the baseline data, but the status of exosuit wearing varied, with participants not wearing exosuits in two studies [12, 16], while another study evaluated participants while both wearing and not wearing an exosuit [15]. The remaining two studies did not explicitly specify whether the participants wore exosuits [13, 17]. The detailed comparator and findings of the included studies are summarized in Table 2.

**Table 2:** Comparator and outcome summaries of the included studies

Study	Comparator (status of exosuit)	Walking ability		Biomechanical outcomes			Other
		Walking speed (m/s)	Walking distance (m)	Peak ankle dorsiflexion angle during swing phase (Degree)	Peak paretic propulsion (%)	Stride length (paretic limb, m)	
Awad et al (2020) [15]	Post-intervention vs baseline (WE)	0.1 ± 0.03 increase (MWS)	NA	NA	NA	NA	NA
	Post-intervention vs baseline (UWE)	0.07 ± 0.03 increase (MWS)					
Shin et al (2022) [16]	Post-intervention vs baseline (UWE)	0.21 ± 0.28 increase (MWS) 0.26 ± 1.1 increase (CWS)	6MWT: 71.5 ± 43.9 increase 2MWT: 18.8 ± 15.1 increase	NA	NA	NA	Fugl-Meyer scale: 1.8 ± 1.5 increase
Poydasheva et al. (2016) [17]	Post-intervention vs baseline (NS)	0.06 increase^a^	NA	NA	NA	NA	Fugl-Meyer scale: 2.1 increase
Awad et al (2017) [19]	Powered exosuit vs unpowered exosuit (WE)	NA	NA	5.33 ± 0.91 increase (treadmill) 4.9 ± 1.1 increase (overground)	11 ± 3 increase (treadmill) 13 increase (overground)	NA	Energy cost: 10 ± 3% reduction
Sloot et al (2022) [20]	WE vs UWE	7% increase	NA	9 increase	8 increase	NA	Energy cost: 11% reduction
Awad et al (2017) [18]	Powered exosuit vs unpowered exosuit (WE)	NA	NA	4.78 increase	NA	0.02 increase	Hip hiking: 27 ± 6% reduction Hip circumduction: 20 ± 5% reduction
Porciuncula et al. (2021) [13]	Post-intervention vs baseline (NS)	0.3 increase (MWS) 0.22 increase (CWS)	6MWT: 59 increase	NA	30 increase^b^ (MWS) 24 increase^b^ (CWS)	0.13 increase (MWS) 0.15 increase (CWS)	Hip hiking: 0.25 cm reduction (MWS), 0.42 cm reduction (CWS) Hip circumduction: 3 cm reduction (MWS), 3.83 cm reduction (CWS)
Porciuncula et al. (2019) [12]	Post-intervention vs baseline (UWE)	0.12 increase	6MWT: 86 increase	NA	10.52 increase	6.7% increase	NA
Kwon et al (2019) [14]	Powered exosuit vs unpowered exosuit vs UWE^c^	NA	NA	10 increase	7.76 increase	NA	NA

m/s = meter/second, m = meter, % = percentage, WE = wear exosuit, MWS = maximal walking speed, NA = not applicable, UWE = unwear exosuit, CWS = comfortable walking speed, 6MWT = 6-min walking test, 2MWT = 2-min walking test, NS = not stated

^a^ Calculated by the data of distance and walking time

^b^ Calculated by the data between the baseline and post-intervention via percentage of body weight

^c^ We only record the result of powered exosuit and unwear exosuit

#### Outcome measures

In primary outcomes, walking speed was measured in seven studies; six of them used 10-m walking test (10MWT) [12, 13, 15–17, 19], one using a 5-min walking test [20]. Walking distance was measured in four studies; three involved a 6-min walking test (6MWT) [12–14] and one used both a 6MWT and two-minute walking test [16]. In biomechanical outcomes, 4 researchers measured peak ankle dorsiflexion angle during swing phase [14, 18–20]. Two [19, 20] measured peak paretic propulsion (measured in percentage of body weight), and four [12–14, 18] measured stride length. In other outcomes, two [16, 17] measured the lower extremity subscale of the Fugl-Meyer scale. Awad et al*.* measured energy cost via an indirect calorimetry system [19], while the other did not report the measurement tool for energy cost [20]. Two measured the degree of hip hiking as well as hip circumduction [13, 18].

### Effects of intervention

#### Primary outcome: walking speed

Increased walking speed was observed in all the reports. Awad et al*.* [15] performed their study with the largest number of participants (*n* = 36). Compared with baseline, a significant increase in maximal walking speed was found after intervention, regardless of wearing an exosuit or not (0.1 ± 0.03 m/s, *P* < 0.001^*^ with an exosuit, and 0.07 ± 0.03 m/s, *P* < 0.01^*^ without). Shin et al*.* [16] analyzed both the maximal walking speed and comfortable walking speed after training, revealing a 0.21 ± 0.28 m/s increase and a 0.26 ± 1.10 m/s increase, respectively. Poydasheva et al*.* [17] found a reduction of 2 s in 10MWT after training (from 19.08 to 17.08 s, *P* = 0.02^*^), with walking speed showing an increase of 0.06 m/s. A case report by Porciuncula et al*.* [13] showed that the maximal walking speed significantly increased by 0.3 m/s (*P* = 0.02^*^) after training, while the comfortable walking speed significantly increased by 0.22 m/s (*P* = 0.04^*^). In the previous case reported by the same author group [12], an increased speed of 0.12 m/s was seen without an exosuit after training. In brief, walking speed increase ranged from 0.06 m/s to 0.3 m/s after intervention.

Sloot et al*.* [20] measured the immediate effect of SREs, and found a speed increase of 7% (*P* = 0.60) when compared with not wearing an exosuit.

#### Primary outcome: walking distance

Three studies recorded walking distance after training. Their distances of 6MWT increased by 71.5 ± 43.9 [16], 59 [13] and 86 m [12], respectively. One [16] study recorded a 2MWT and showed an increase of 18.8 ± 15.1 m.

#### Biomechanical outcome: peak ankle dorsiflexion angle during swing phase

Four studies evaluated the immediate effect of the exosuit. Awad et al*.* [19] found that with a powered exosuit, the peak ankle dorsiflexion angle during swing phase had significantly increased by 5.33 ± 0.91° on a treadmill (from -1.85 ± 1.98° to 3.49 ± 1.52°, *P* < 0.001^*^) and by 4.9 ± 1.1° on the ground (from -4.13 ± 1.82° to 30.74 ± 1.51°, *P* < 0.002^*^), when compared with an unpowered exosuit.

Similarly, another study involving an actuated exosuit [Awad et al*.* [18]] also revealed a significant improvement of 4.78° (from 0.52 ± 2.06° to 4.26 ± 1.84°, *P* = 0.002^*^). In addition, Sloot et al*.* [20] and Kwon et al*.* [14] compared the peak ankle dorsiflexion angle during swing phase between the powered exosuit and barefoot walking, and reported a 9° increase (*P* = 0.003^*^) and 10° increase, respectively.

#### Biomechanical outcome: peak paretic propulsion

Porciuncula et al*.* [13] found that after training, both at maximal walking speed and comfortable walking speed, the median peak paretic propulsion increased by approximately 30% (from 15.22%BW (interquartile: 2.82) to 19.85%BW (interquartile: 1.88, *P* = 0.02^*^) and 24% (from 11.43%BW (interquartile: 1.43) to 14.23%BW (interquartile: 0.98), *P* = 0.04^*^), respectively. In another article, Porciuncula et al*.* [12] reported a 10.52% increase in propulsion force after intervention.

As for the immediate effect, Awad et al*.* [19] compared results of the powered and unpowered exosuits. They found that the peak paretic propulsion on the treadmill with the powered exosuit had increased from 11.39 ± 2.31%BW to 12.66 ± 2.35% BW, which was a significant difference in percentage (11 ± 3%, *P* = 0.009^*^). While walking overground, the powered exosuit showed a 13% increase from 10.3 ± 0.60%BW to 11.6 ± 0.60%BW (*P* = 0.053*). Sloot et al*.* [20] also found an 8% increase (*P* = 0.03^*^), with Kwon et al*.* [14] reporting a 7.76% increase with an exosuit when compared with not wearing an exosuit.

#### Biomechanical outcome: stride length

After intervention, Porciuncula et al*.* [13] found an increase of 0.13 m (*P* = 0.002*) at maximal walking speed and an increase of 0.15 m (*P* = 0.04*) in comfortable walking speed. Porciuncula et al*.* [12] also reported an increase of 6.7%.

#### Other outcome: Fugl-Meyer scale

Two studies reported increases in the Fugl-Meyer scale after training, with a 1.8 ± 1.5 increase seen in the study performed by Shin et al*.* [16] and 2.1 (*P* = 0.113846) witnessed by Poydasheva et al*.* [17].

#### Other outcomes: energy costs

Powered exosuits successfully decreased energy costs by 10 ± 3% (P = 0.009*) when compared with unpowered exosuits in a study performed by Awad et al*.* [19]. A reduction of 11% in energy costs when wearing an exosuit compared to not wearing an exosuit (P = 0.31) was also found by Sloot et al*.* [20].

#### Other outcomes: hip hiking

After training, Porciuncula et al*.* [13] reported hip hiking decreased 0.25 cm (*P* = 0.38) at maximal walking speed and 0.42 cm (*P* = 1.0) at comfortable walking speed. The immediate effect reported by Awad et al*.* [18] was a 27 ± 6% (*P* = 0.004*) drop in hip hiking with a powered exosuit when compared with an unpowered exosuit.

#### Other outcomes: hip circumduction

After training, Porciuncula et al*.* [13] reported that hip circumduction decreased 3 cm (*P* = 0.52) at maximal walking speed and 3.83 cm (*P* = 0.004*) at a comfortable walking speed. The immediate effect reported by Awad et al*.* [18] was a 20 ± 5%(p = 0.004*) decrease with a powered exosuit when compared with an unpowered exosuit.

#### Summary on the effect of intervention

In summary, SREs improved both walking speed and distance after training (one study focused on the immediate effect of walking speed but showed no significance [20]). In addition, biomechanical outcomes, such as peak ankle dorsiflexion angle during swing phase, peak paretic propulsion and stride length, all improved in both the immediate and the training effect. Other outcomes including the Fugl-Meyer scale and energy cost also improved. Decreased hip circumduction was also noticed after using SREs.

## Discussion

In this comprehensive systematic review, we found that SREs improve walking speed, walking distance, biomechanical variables of the gait cycle (including the peak ankle dorsiflexion angle during swing phase, peak paretic propulsion and stride length), while reducing the compensatory gait mechanisms. To the best of our knowledge, our study is the first systematic review to assess and summarize both the immediate and training effects of the SRE on ambulation ability in stroke patients.

### Immediate effect: biomechanical variables of gait cycle

Four studies focused on the immediate effect of the exosuit [14, 18–20]. Most of them found improvements in ground clearance and peak paretic propulsion but not with walking speed. The improvement of the ankle dorsiflexion angle during swing phase ranged from 4.78 to 10 degrees. Grimmer et al*.* summarized two different mechanisms regarding the assistance techniques for generating the force to the ankle joint during gait cycle: namely the ankle moment inspired technique and the ankle positive power inspired technique [21]. Similar peak positive exosuit push-off power was noted when comparing the two mechanisms, with an average of 1.31 Nm/kg being seen [21], which was similarly mentioned in studies by both Awad et al*.* [19] and Kwon et al*.* [14]. As for the peak paretic propulsion percentage, the rising extent varied between 7.73 and 13%. SREs likely improve paretic limb function, while also reducing energy expenditure during hemiparetic gait by strengthening the paretic plantar flexors during mid-to-late stance and the paretic dorsiflexors during swing phase [19]. Compared with conventional ankle foot orthosis (AFO), which restricts ankle range of motion and impairs the generation of forward propulsion during walking, SRE could reduce the energy cost of walking [22].

### Training effect: walking speed, walking distance and biomechanical variables of gait cycle

The other 5 trials studied the training effects of the exosuit [12, 13, 15–17], where the frequency of intervention ranged from 5 to 18 sessions and the follow-up period varied from 2 weeks to 2 months. All studies found increases in walking speed and most of them also showed improved walking distances. The increase in walking speed ranged from 0.06 to 0.26 m/s, with minimal clinically important difference (MCID) value of 0.05 m/s being reported earlier[23, 24]. As for walking distance, measured at 6MWT, improvements ranged from 59 to 86 m with a MCID threshold of 34.4 m being stated [25]. All improvements exceeded the MCID values, indicating that the improvement achieved not only statistical significance, but also clinical importance. Similarly, biomechanical outcomes, in terms of ankle dorsiflexion angle during swing phase and peak paretic propulsion, also significantly improved, with the range of training effect being from 4.78 to 10 degrees and 0.52 to 30%, respectively. As most wearable exoskeletons do not have actuators over the ankle joint, no information is available regarding MCID values in the related biomechanical variables discussed in this paper [26]. It is well known that neuroplastic change during locomotor neurorehabilitation is crucial for functional improvement [27]. Kim et al*.* conducted a randomized controlled trial on patients with post-stroke hemiparesis, comparing end-effector robot-assisted gait training (E-RAGT) and body-weight-supported treadmill training (BWST). They found greater activations occurring in the primary sensorimotor cortex, supplementary motor area and premotor cortex of the affected hemisphere in the E-RAGT group only, although with no significant inter-group difference being seen [27]. Presumably, similar neuroplastic changes may also develop after the SRE training.

In summary, regarding training effects, SREs improved both walking speed and walking distance, and both exceeded the MCID value. In addition, biomechanical outcomes, including ankle dorsiflexion angle during swing phase and peak paretic propulsion also improved, although no MCID value was mentioned in the previous papers. We believe that this may be related to neuroplastic changes after SRE training.

### Compared with the exoskeleton systems

Although exoskeletal wearable robotic devices are considered effective according to the current rehabilitation guidelines, their cumbersomeness and rigid structures limit their overall functionality and movement ranges. Rodríguez-Fernández et al*.* summarized 25 commercially available exoskeletons for stroke patients, with the mean device weights being 8.90 ± 7.48 kg [26]. In contrast, the exosuits in the studies evaluated in this review only weighed 0.9–5 kg. However, it is this rigid actuated device that could help non-ambulatory subacute stroke patients engage early in high-intensity rehabilitation programs [28]. Previous studies have investigated the efficacy of the exoskeleton systems mainly regarding walking ability (6MWT or time up to go test) and balance function (Berg Balance Scale) [9, 29]. Wright et al*.* revealed their walking benefits after a 10-week overground robotic assisted gait training, which used the exoskeleton for ≥ 30 min/day. Here, the 6MWT test showed improvements from 135 ± 81 m to 158 ± 93 m (P ≤ 0.001), as well as better coordination on the Berg Balance scale (p ≤ 0.01) [8]. Comparing improvements in 6MWT, the three included studies all revealed greater increases than that found in Wright’s 2021 trial [8] (71.5 ± 43.9 m in the Shin et al*. *study [16], 59 m in the Porciuncula et al*. *study [13], and 86 m in the Porciuncula et al*. *study [12]). However, baseline characteristics of their patients were different, with better ambulation ability being evident prior to intervention in the exosuit group (6MWT value: 392.8 ± 57.5 m in Shin et al*.* [16] and 435 m in Porciuncula et al*.* [13]), suggesting that the exosuit may be less suitable for patients with severe motor impairments, which is consistent with previous consensuses [19, 30].

## Limitations

The SRE is a novel and developing technology, therefore, few studies have been published. Current literature centering on the technological features of the exosuit are not limited to stroke patients. Several limitations to the technology do exist, however. First, the present literature we reviewed involved mostly studies adopting a research design ranked low on the hierarchy of scientific evidence (i.e., case studies and non-comparative interventional studies), so they failed to provide strong conclusions regarding the effects of SREs. The diversity of our selected articles (e.g., use of numerous robotic exosuits having different attributes; recruitment of small and heterogeneous samples; adoption of numerous intervention protocols, and selection of diverse outcome measures) further limits the strengths of our conclusions. Second, 7 of the 9 enrolled studies came from the same research team (with the exception of Poydasheva et al*.* [17] and Kwon et al. [14]), with most studies being centered on American populations. Although we had carefully reviewed the study groups and excluded overlapping ones, there still remained a possibility for potential bias. Third, the use of assisted devices (e.g., walking canes) also contributed to the variable outcomes during the gait cycle. Only 4 of the 9 studies provided such relevant information. Fourth, it is well known that there is no shortcut to neurorehabilitation and that it requires gradually rhythmic, repetitive and concentrated practice. It has been suggested that for stroke patients, at least 300–500 repetitions are required for the recovery of lower limb motor function and neuroplasticity [4, 31]. The intervention periods in our included studies were all too short in this regard. Besides, SRE is primarily designed for personal daily use at home. Therefore, trials with longer intervention periods should be conducted in the future in order to provide stronger evidence for support of SRE use. Finally, all studies in this review were either non-comparative interventional studies or case reports. Due to the lack of control groups, one cannot rule out time effect. As participants in these studies were all stroke patients in chronic phase, they seldom displayed radical neurological change during their study period, therefore, the time effect was likely small.

Future studies should be performed which enroll more patients, have a more quasi-experimental design and provide more subgroup analysis, in order to better strengthen the evidence level given the diversity of the patient populations.

## Conclusion

Effects of SREs on ambulation ability in stroke patients can be summated through the increased walking speed and distance covered by those who use them, which in turn improves one’s mobility potential. With regard to biomechanical properties, walking training while wearing an SRE enhances ankle dorsiflexion angle during swing phase and peak paretic propulsion, while also reducing abnormal gait patterns such as hip hiking and circumduction gait. However, the lack of relevant studies limits the generalizability and reduce the confidence in the interpretation. More controlled studies of a better quality and higher evidence level are still required in order to reach a more solid conclusion on the impact which SREs have on ambulation training in stroke patients.

## Methods

The protocol was registered in advance on the International Prospective Register of Systematic Reviews (PROSPERO CRD42022356458). This systematic review was conducted in accordance with the Preferred Reporting Items for Systematic Reviews and Meta-Analyses (PRISMA 2020) statement [32]. The PRISMA 2020 Checklist is shown in Additional File 1: Table S1.

### Search strategy and selection criteria

Five databases (PubMed, Embase, Cochrane Library, Web of Science, and the Physiotherapy Evidence Database [PEDro]) were all searched from the point of their inceptions. Different combinations of the following keywords and their equivalents were applied: ‘stroke’, ‘exosuit’, ‘myosuit’, ‘soft exoskeleton’, and ‘soft robotic’. The detailed search strategy is shown in Additional File 2: Table S2. Two investigators (Y. C. C. and Y. L. T.) conducted the initial literature screening by reviewing titles and abstracts without including any filters.

Studies were included if they met the following criteria: (1) population: patients with post-stroke hemiparesis; (2) intervention: gait training with an SRE or similar interventional device (defined as weight-bearing relies on the user’s skeletal structure alone and excluded the exoskeletons with rigid external structure); (3) control: not stipulated; and (4) outcomes: walking ability (such as walking speed and walking distance), biomechanical outcome (such as peak ankle dorsiflexion angle during swing phase, peak paretic propulsion and stride length), as well as any other possible ambulation-related outcomes. Studies were excluded if their data were inaccessible. The detailed reasons for exclusion of the studies are disclosed in Additional File 3: Table S3. Disagreements between the two investigators were discussed with a third investigator (L. J. O. Y.) to reach a consensus.

### Assessment of the characteristics of the trials

#### Methodological quality of the trials

Two independent investigators (Y. C. C. and Y. L. T.) appraised the quality of the included trials. Any disagreement between the two was resolved by a third reviewer (L. J. O. Y.). The risk of bias in the randomized controlled trials (RCTs) was evaluated using the ‘risk of bias tool 2.0 (RoB 2.0)’ [33]. This assessment contains 5 domains: the randomization process, deviation from the intended intervention, missing outcome data, outcome measurement, and selective outcome reporting. This study used the risk of bias in non-randomized studies of interventions (ROBINS-I) in order to assess the risk of bias in non-RCTs [34]. Single-group cohort studies, case serials and case reports were considered low quality of evidence if they were unable to be assessed via the aforementioned tools.

However, after searching, we found that only single-group studies and case reports met the inclusion criteria, and were, therefore, not suitable to be assessed with RoB 2.0 and ROBINS-I.

#### Inclusion criteria

Trials were considered eligible if they had studied the effects of SREs and any similar devices with regard to ambulation ability in patients with post-stroke hemiparesis. The common inclusion criteria for the patients were being of age > 18 years and able to follow simple commands. Studies were excluded if their results were not mentioned in the outcome measures or if their data were inaccessible.

#### Outcome measures

The primary outcomes of this review were walking speed (m/s) and walking distance (m). The secondary outcomes were biomechanical factors, including peak ankle dorsiflexion angle during swing phase and peak paretic propulsion and stride length, which are measured in degree/percentage/meter when appropriate.

### Qualitative data synthesis

The names of the authors, years of publication, locations of the study, basic data of participants (age, stroke latency), types of device, intervention protocol, and outcomes were all extracted. Data extraction was performed independently by two authors (Y. L. T. and L. J. O. Y.). Discrepancies were resolved after discussion with a third author (Y. C. C.). For missing data or uncertain issues, we corresponded with the authors of the study via email. Studies were excluded from the data analysis if data were inaccessible, or if the authors were not responding. All findings from the included studies were narrated in detail.

### Supplementary Information

**Additional file 1: Table S1**. PRISMA 2020 Checklist.

**Additional file 2: Table S2.** The detailed search strategy.

**Additional file 3: Table S3. **Reasons for exclusion (n=96).

## Data Availability

The datasets used and/or analyzed in the current study are available upon request from the corresponding author. The original papers presented in the study are included in the Article/Additional files. Further inquiries can be directed to the corresponding author.
