# Supplementary material for: Effects of soft robotic exosuit on ambulation ability in stroke patients: a systematic review
Source: Biomed Eng Online. 2023 Sep 5;22:88. doi: 10.1186/s12938-023-01150-7 (PMC10478336; doi:10.1186/s12938-023-01150-7)
Supplement: Supplementary file 2 — Additional file 2: Table S2. The detailed search strategy. [file 12938_2023_1150_MOESM2_ESM.docx]

**Additional file 2: Table S2**

| **Electronic search strategy** | | |
| --- | --- | --- |
| **PubMed search** | | |
| Population | #1 | ("Stroke"[Mesh] OR "Stroke, Lacunar"[Mesh] OR "National Institute of Neurological Disorders and Stroke (U.S.)"[Mesh] OR "Hemorrhagic Stroke"[Mesh] OR "Embolic Stroke"[Mesh] OR "Thrombotic Stroke"[Mesh] OR "Ischemic Stroke"[Mesh] OR "Stroke Rehabilitation"[Mesh] OR "Infarction, Posterior Cerebral Artery"[Mesh] OR "Brain Stem Infarctions"[Mesh] OR "Infarction, Middle Cerebral Artery"[Mesh] OR "Infarction, Anterior Cerebral Artery"[Mesh] OR "Anterior spinal artery stroke") |
| Intervention | #2 | ("Myosuit" OR "Exosuit" OR "Soft exoskeleton" OR "Soft robotic exosuit" OR "Wearable robotic" OR "Soft robotic suit" OR "Soft wearable robot" OR "Soft exosuit" OR "Soft Robotic" OR ("Robotic" AND "Soft")) |
| Filters |  | None |
| Search algorithm | #3 | #1 AND #2 |
| **Cochrane Library Databases search** (Title, Abstract, Keywords) | | |
| Population | #1 | ("Stroke" OR "Hemorrhagic Stroke" OR "Embolic Stroke" OR "Thrombotic Stroke" OR "Ischemic Stroke") |
| Intervention | #2 | ("Myosuit" OR "Exosuit" OR "Soft exoskeleton" OR "Soft robotic exosuit" OR "Wearable robotic" OR "Soft robotic suit" OR "Soft wearable robot" OR "Soft exosuit" OR "Soft Robotic" OR ("Robotic" AND "Soft")) |
| Filters |  | None |
| Search algorithm | #3 | #1 AND #2 |
| **Embase search** (Title, Abstract, Author keywords) | | |
| Population | #1 | ("Stroke" OR "Hemorrhagic Stroke" OR "Embolic Stroke" OR "Thrombotic Stroke" OR "Ischemic Stroke") |
| Intervention | #2 | ("Myosuit" OR "Exosuit" OR "Soft exoskeleton" OR "Soft robotic exosuit" OR "Wearable robotic" OR "Soft robotic suit" OR "Soft wearable robot" OR "Soft exosuit" OR "Soft Robotic" OR ("Robotic" AND "Soft")) |
| Filters | #3 | (Trials) |
| Search algorithm | #4 | #1 AND #2 AND #3 |
| **Web of Science search** | | |
| Population | #1 | ("Stroke" OR "Hemorrhagic Stroke" OR "Embolic Stroke" OR "Thrombotic Stroke" OR "Ischemic Stroke") |
| Intervention | #2 | ("Myosuit" OR "Exosuit" OR "Soft exoskeleton" OR "Soft robotic exosuit" OR "Wearable robotic" OR "Soft robotic suit" OR "Soft wearable robot" OR "Soft exosuit" OR "Soft Robotic" OR ("Robotic" AND "Soft")) |
| Filters |  | None |
| Search algorithm | #3 | #1 AND #2 |
| **PEDro search** | | |
| Abstract and Title | #1 | Myosuit |
| Abstract and Title | #2 | Exosuit |
| Abstract and Title | #3 | Soft exoskeleton |
| Abstract and Title | #4 | Soft robotic exosuit |
| Abstract and Title | #5 | Wearable robotic |
| Abstract and Title | #6 | Soft robotic suit |
| Abstract and Title | #7 | Soft wearable robot |
| Abstract and Title | #8 | Soft exosuit |
| Abstract and Title | #9 | Soft Robotic |
| Abstract and Title | #10 | Robotic, Soft |
| Search algorithm | #11 | #1 AND #2 AND #3 AND #4 AND #5 AND #6 AND #7 AND #8 AND #9 AND #10 |
