# Supplementary material for: Effects of soft robotic exosuit on ambulation ability in stroke patients: a systematic review
Source: Biomed Eng Online. 2023 Sep 5;22:88. doi: 10.1186/s12938-023-01150-7 (PMC10478336; doi:10.1186/s12938-023-01150-7)
Supplement: Supplementary file 3 — Additional file 3: Table S3. Reasons for exclusion (n=96). [file 12938_2023_1150_MOESM3_ESM.docx]

**Additional file 3** Reasons for exclusion (n=96).

| Study | Title of the article | Additional notes |
| --- | --- | --- |
| 1. Proulxi 2020 | Perceived Usability and Acceptability of a Soft Robotic Glove for Rehabilitation of Adults With Hand Hemiparesis: A Mixed-Method Study Among Occupational Therapists in Stroke Rehabilitation | Intervention didn’t meet criteria (use Soft Robotic Glove) |
| 2. Asgher 2021 | Motor Training Using Mental Workload (MWL) With an Assistive Soft Exoskeleton System: A Functional Near-Infrared Spectroscopy (fNIRS) Study for Brain–Machine Interface (BMI) | Intervention didn’t meet criteria (use Robotic Exoskeleton glove) |
| 3. Biggar 2016 | Design and Evaluation of a Soft and Wearable Robotic Glove for Hand Rehabilitation | Intervention didn’t meet criteria (use Soft Robotic Glove) |
| 4.Cheng 2020 | Brain-Computer Interface-based Soft Robotic Glove Rehabilitation for Stroke | Intervention didn’t meet criteria (use Soft Robotic Glove) |
| 5. Buesing 2015 | Effects of a wearable exoskeleton stride  management assist system (SMA®) on  spatiotemporal gait characteristics in  individuals after stroke: a randomized  controlled trial | Intervention didn’t meet criteria (use exoskeleton) |
| 6. Bützer 2021 | Fully Wearable Actuated Soft Exoskeleton for Grasping Assistance in Everyday Activities | Intervention didn’t meet criteria (use Soft Robotic Glove) |
| 7. Cappello 2018 | Assisting hand function after spinal cord  injury with a fabric-based soft robotic glove | Intervention didn’t meet criteria (use Soft Robotic Glove) |
| 8. Chang 2018 | Exoskeleton-assisted gait training to  improve gait in individuals with spinal cord injury: a pilot randomized study | Intervention didn’t meet criteria (use exoskeleton) |
| 9. Chen 2022 | Soft Exoskeleton With Fully Actuated Thumb Movements for Grasping Assistance | Intervention didn’t meet criteria (use Soft Robotic Glove) |
| 10. Correia 2020 | Improving Grasp Function after Spinal Cord Injury with a Soft Robotic Glove | Intervention didn’t meet criteria (use Soft Robotic Glove) |
| 11. Cuillo 2020 | A Novel Soft Robotic Supernumerary Hand for Severely Affected Stroke Patients | Intervention didn’t meet criteria (use Soft Robotic Glove) |
| 12. Delph II 2013 | A Soft Robotic Exomusculature Glove with Integrated sEMG Sensing for Hand Rehabilitation | Intervention didn’t meet criteria (use Soft Robotic Glove) |
| 13. Durandau 2019 | Voluntary control of wearable robotic  exoskeletons by patients with paresis via  neuromechanical modeling | Intervention didn’t meet criteria (use exoskeleton) |
| 14. Escalona 2020 | Wearable exoskeleton control modes selected during overground walking affect muscle synergies in adults with a chronic incomplete spinal cord injury | Intervention didn’t meet criteria (use exoskeleton) |
| 15. Gagnon 2017 | How does wearable robotic exoskeleton affect overground walking  performance measured with the 10-m and six-minute walk tests after a basic  locomotor training in healthy individuals? | Intervention didn’t meet criteria (use exoskeleton) |
| 16. Gerez 2020 | A Hybrid, Wearable Exoskeleton Glove Equipped With Variable Stiffness Joints, Abduction Capabilities, and a Telescopic Thumb | Intervention didn’t meet criteria (use Soft Robotic Glove) |
| 17. Guo 2022 | SSVEP-Based Brain Computer Interface  Controlled Soft Robotic Glove for Post-Stroke Hand Function Rehabilitation | Intervention didn’t meet criteria (use Soft Robotic Glove) |
| 18. Haghshenas-Jaryani 2019 | Soft Robotic Bilateral Hand Rehabilitation System for Fine Motor Learning* | Intervention didn’t meet criteria (use Soft Robotic Glove) |
| 19. Heung 2019 | Robotic Glove with Soft-Elastic Composite Actuators  for Assisting Activities of Daily Living | Intervention didn’t meet criteria (use Soft Robotic Glove) |
| 20. Hunt 2019 | A Soft-Robotic Harbor Porpoise Pectoral Fin Driven by Coiled Polymer Actuators as Artificial Muscles | Intervention didn’t meet criteria (use Soft Robotic Glove) |
| 21. Hussain 2017 | A soft supernumerary robotic finger and mobile arm support for grasping compensation and hemiparetic upper limb rehabilitation | Intervention didn’t meet criteria (use Soft Robotic Glove) |
| 22. Jeong 2019 | Design of Shape Memory Alloy-Based Soft Wearable Robot for Assisting Wrist Motion | Intervention didn’t meet criteria (use Soft Robotic Glove) |
| 23. Jeong 2022 | Soft Wearable Robot With Shape Memory Alloy (SMA)-Based Artificial Muscle for Assisting With Elbow Flexion and Forearm Supination/Pronation | Intervention didn’t meet criteria (use Soft Robotic Glove) |
| 24. Jumphoo 2021 | Soft Robotic Glove Controlling Using Brainwave Detection for Continuous Rehabilitation at Home | Intervention didn’t meet criteria (use Soft Robotic Glove) |
| 25. Kang 2019 | Exo-Glove Poly II: A Polymer-Based Soft Wearable Robot for the Hand with a Tendon-Driven Actuation System | Intervention didn’t meet criteria (use Soft Robotic Glove) |
| 26. Kelvin 2019 | Design of 3D printed soft robotic hand for stroke rehabilitation and daily activities assistance | Intervention didn’t meet criteria (use Soft Robotic Glove) |
| 27. Kim 2013 | Incorporating a Wearable Upper Extremity Robotics Device into Daily  Activities at Home: A Case Series | Intervention didn’t meet criteria (use Soft Robotic Glove) |
| 28. Kim 2013 | Kinematic Data Analysis for Post-Stroke Patients Following Bilateral Versus Unilateral Rehabilitation With an Upper Limb Wearable Robotic System | Intervention didn’t meet criteria (use Soft Robotic Glove) |
| 29. Kim 2020 | Joint Angle Estimation of a Tendon-Driven Soft Wearable Robot through a Tension and Stroke Measurement | Intervention didn’t meet criteria (use Soft Robotic Glove) |
| 30. Klug 2019 | An Anthropomorphic Soft Exosuit for Hand Rehabilitation | Intervention didn’t meet criteria (use Soft Robotic Glove) |
| 31. Koh 2017 | Design of a Soft Robotic Elbow Sleeve with Passive and Intent-Controlled Actuation | Intervention didn’t meet criteria (use Soft Robotic Glove) |
| 32. Koyama 2022 | Wearable Power-Assist Locomotor for Gait Reconstruction in Patients With Spinal Cord Injury: A Retrospective Study | Intervention didn’t meet criteria (use exoskeleton) |
| 33. Lee 2019 | Training for Walking Efficiency With a Wearable Hip-Assist Robot in Patients With Stroke A Pilot Randomized Controlled Trial | Intervention didn’t meet criteria (use exoskeleton) |
| 34. Lessard 2018 | A Soft Exosuit for Flexible Upper-Extremity Rehabilitation | Intervention didn’t meet criteria (use Soft Robotic Glove) |
| 35. Li 2015 | Improved walking ability with wearable  robot-assisted training in patients suffering chronic stroke | Intervention didn’t meet criteria (use exoskeleton) |
| 36. Li 2018 | Bio-inspired upper limb soft exoskeleton to reduce stroke-induced complications | Intervention didn’t meet criteria (use Soft Robotic Glove) |
| 37. Low 2019 | Effect of a Soft Robotic Sock Device on Lower Extremity Rehabilitation Following Stroke: A Preliminary Clinical Study With Focus on Deep Vein Thrombosis Prevention | Intervention didn’t meet criteria (use Soft Robotic sock) |
| 38. McCall 2021 | High Compliance Pneumatic Actuators to Promote Finger Extension in Stroke Survivors | Intervention didn’t meet criteria (use Soft Robotic Glove) |
| 39. Molteni 2017 | Wearable robotic exoskeleton for overground gait training in sub-acute and chronic hemiparetic stroke patients: preliminary results | Intervention didn’t meet criteria (use exoskeleton) |
| 40. Burns 2019 | Myoelectric Control of a Soft Hand Exoskeleton Using Neural Networks and Kinematic Synergies | Intervention didn’t meet criteria (use Soft Robotic Glove) |
| 41. Byl 2013 | Chronic stroke survivors achieve comparable outcomes following virtual task specific repetitive training guided by a wearable robotic orthosis (UL-EXO7)  and actual task specific repetitive training guided by a physical therapist | Intervention didn’t meet criteria (use Soft Robotic Glove) |
| 42. Nasrallah 2021 | Effect of proprioceptive stimulation using a soft robotic glove on motor activation and brain connectivity in stroke survivors | Intervention didn’t meet criteria (use Soft Robotic Glove) |
| 43. Nazari 2021 | A Compact and Lightweight Rehabilitative Exoskeleton to  Restore Grasping Functions for People with Hand Paralysis | Intervention didn’t meet criteria (use Soft Robotic Glove) |
| 44. Noronha 2022 | Soft, Lightweight Wearable Robots to Support the Upper Limb in Activities of Daily Living: A Feasibility Study on Chronic Stroke Patients | Intervention didn’t meet criteria (use Soft Robotic Glove) |
| 45. Nuckols 2019 | Proof of Concept of Soft Robotic Glove for Hand Rehabilitation in Stroke Survivors | Intervention didn’t meet criteria (use Soft Robotic Glove) |
| 46. O'Neill 2020 | Inflatable Soft Wearable Robot for Reducing Therapist Fatigue During Upper Extremity Rehabilitation in Severe Stroke | Intervention didn’t meet criteria (use Soft Robotic Glove) |
| 47. Osuagwu 2020 | Home-based rehabilitation using a soft  robotic hand glove device leads to  improvement in hand function in people  with chronic spinal cord injury:a pilot study | Intervention didn’t meet criteria (use Soft Robotic Glove) |
| 48. Palmcrantz 2020 | Factors affecting the usability of an assistive soft robotic glove after stroke or multiple sclerosis | Intervention didn’t meet criteria (use Soft Robotic Glove) |
| 49. Polygerinos 2015 | Soft robotic glove for combined assistance and at-home rehabilitation | Intervention didn’t meet criteria (use Soft Robotic Glove) |
| 50. Prange 2017 | Applying a soft-robotic glove as assistive device and training tool with games to support hand function after stroke: preliminary results on feasibility and potential clinical impact | Intervention didn’t meet criteria (use Soft Robotic Glove) |
| 51. Proulx 2021 | Occupational therapists’ evaluation of the  perceived usability and utility of wearable soft robotic exoskeleton gloves for hand function rehabilitation following a stroke | Intervention didn’t meet criteria (use Soft Robotic Glove) |
| 52. Qiu 2020 | Synergistic Immediate Cortical Activation on Mirror Visual Feedback Combined With a Soft Robotic Bilateral Hand Rehabilitation System: A Functional Near Infrared Spectroscopy Study. | Intervention didn’t meet criteria (use Soft Robotic Glove) |
| 53. Radder 2020 | The effect of a wearable soft-robotic glove on motor function and functional performance of older adults | Intervention didn’t meet criteria (use Soft Robotic Glove) |
| 54. Radder 2018 | Feasibility of a wearable soft-robotic glove to support impaired hand function in stroke patients | Intervention didn’t meet criteria (use Soft Robotic Glove) |
| 55. Radder 2019 | Home rehabilitation supported by a wearable soft-robotic device for improving hand function in older adults: A pilot randomized controlled trial | Intervention didn’t meet criteria (use Soft Robotic Glove) |
| 56. Rahman 2015 | Development of a whole arm wearable robotic exoskeleton for rehabilitation and to assist upper limb movements | Intervention didn’t meet criteria (use robotic exoskeleton Glove) |
| 57. Randazzo 2018 | mano: A Wearable Hand Exoskeleton for Activities of Daily Living and Neurorehabilitation | Intervention didn’t meet criteria (use robotic exoskeleton Glove) |
| 58. Ren 2017 | Developing a wearable ankle rehabilitation robotic device for in-bed acute stroke rehabilitation | Intervention didn’t meet criteria (use exoskeleton) |
| 59. Rudd 2019 | A Low-Cost Soft Robotic Hand Exoskeleton for Use in Therapy of Limited Hand–Motor Function | Intervention didn’t meet criteria (use robotic exoskeleton Glove) |
| 60. Ryser 2017 | Fully Embedded Myoelectric Control for a Wearable Robotic Hand Orthosis | Intervention didn’t meet criteria (use Soft Robotic Glove) |
| 61. Shi 2020 | Verification of Finger Joint Stiffness Estimation Method With Soft Robotic Actuator | Intervention didn’t meet criteria (use Soft Robotic Glove) |
| 62. Shi 2021 | Effects of a Soft Robotic Hand for Hand Rehabilitation in Chronic Stroke Survivors | Intervention didn’t meet criteria (use Soft Robotic Glove) |
| 63. Tanabe 2013 | Wearable Power-Assist Locomotor (WPAL) for supporting upright walking in persons with paraplegia | Intervention didn’t meet criteria (use exoskeleton) |
| 64. Tang 2021 | Model-based online learning and adaptive control for a ‘‘human-wearable soft robot’’ integrated system | Intervention didn’t meet criteria (use Soft Robotic Glove) |
| 65. Tang 2022 | Probabilistic Model-Based Learning Control of a Soft Pneumatic Glove for Hand Rehabilitation | Intervention didn’t meet criteria (use Soft Robotic Glove) |
| 66. Thimabut 2022 | Effectiveness of a Soft Robotic Glove to Assist Hand Function in Stroke Patients: A Cross-Sectional Pilot Study | Intervention didn’t meet criteria (use Soft Robotic Glove) |
| 67. Ommeren 2019 | Quantifying upper extremity performance with and without assistance of a soft-robotic glove in elderly patients: A kinematic analysis | Intervention didn’t meet criteria (use Soft Robotic Glove) |
| 68. Vélez-Guerrero 2021 | Design, Development, and Testing of an Intelligent Wearable Robotic Exoskeleton Prototype for Upper Limb Rehabilitation | Intervention didn’t meet criteria (use Soft Robotic Glove) |
| 69. Vélez-Guerrero 2022 | Assessment of the Mechanical Support Characteristics of a Light and Wearable Robotic Exoskeleton Prototype Applied to Upper Limb Rehabilitation | Intervention didn’t meet criteria (use Soft Robotic Glove) |
| 70. Washabaugh 2018 | A wearable resistive robot facilitates  locomotor adaptations during gait | Intervention didn’t meet criteria (use exoskeleton) |
| 71. Wong 2012 | A wearable robotic knee orthosis for gait training: a case-series of hemiparetic stroke survivors | Intervention didn’t meet criteria (use exoskeleton) |
| 72. Yap 2016 | A Low-Profile Soft Robotic Sixth-Finger for Grasp Compensation in Hand-Impaired Patients1 | Intervention didn’t meet criteria (use Soft Robotic Glove) |
| 73. Yap 2016 | Design of a Soft Robotic Glove  for Hand Rehabilitation of Stroke  Patients With Clenched Fist Deformity  Using Inflatable Plastic Actuators | Intervention didn’t meet criteria (use Soft Robotic Glove) |
| 74. Yap 2017 | A Fully Fabric-Based Bidirectional Soft  Robotic Glove for Assistance and  Rehabilitation of Hand Impaired Patients | Intervention didn’t meet criteria (use Soft Robotic Glove) |
| 75. Yap 2017 | A Magnetic Resonance Compatible Soft  Wearable Robotic Glove for Hand  Rehabilitation and Brain Imaging | Intervention didn’t meet criteria (use Soft Robotic Glove) |
| 76. Yap 2017 | Design and Preliminary Feasibility Study of a Soft Robotic Glove for Hand Function Assistance in Stroke Survivors | Intervention didn’t meet criteria (use Soft Robotic Glove) |
| 77. Yeung 2021 | Efects of wearable ankle robotics for stair  and over-ground training on sub-acute stroke: a randomized controlled trial | Intervention didn’t meet criteria (use exoskeleton) |
| 78. Yi 2018 | A Three-Dimensional-Printed Soft Robotic Glove With Enhanced Ergonomics and Force Capability | Intervention didn’t meet criteria (use Soft Robotic Glove) |
| 79. Wang 2022 | Soft Exoskeleton Mimics Human Cough  for Assisting the Expectoration Capability of SCI Patients | Intervention didn’t meet criteria (use Soft exoskeleton for lung) |
| 80. Gasperini 2018 | Recovery of gait function with a wearable powered exoskeleton in sub-acute stroke patients using SEMG for fine tuning: Preliminary results | Intervention didn’t meet criteria (use exoskeleton) |
| 81. Kim 2021 | High-Degrees of Freedom Soft Robotic Glove for Restoring Versatile and Comfortable Manipulation | Intervention didn’t meet criteria (use Soft Robotic Glove) |
| 82. Abbasi 2017 | Lightweight Exosuit Could Help  Patients Walk After Stroke | News |
| 83. Chen 2018 | Ankle passive and active movement training in children with acute brain injury using a wearable robot | Participants didn’t meet criteria ( traumatic brain injury children) |
| 84. Baye-Wallace 2022 | Entrainment During Human Locomotion Using a Lightweight Soft Robotic Hip Exosuit (SR-HExo) | Participants didn’t meet criteria (healthy subjects) |
| 85. Haufe 2020 | Activity-based training with the Myosuit:  a safety and feasibility study across diverse gait disorders | Participants didn’t meet criteria (diverse motor problems) |
| 86. Jarrett 2017 | Robust Control of a Cable-Driven Soft  Exoskeleton Joint for Intrinsic Human-Robot Interaction | Participants didn’t meet criteria (healthy subjects and cerebral palsy) |
| 87. Lee 2017 | Gait performance and foot pressure  distribution during wearable robot-assisted gait in elderly adults | Participants didn’t meet criteria (healthy subjects) |
| 88. Swaminathan 2021 | Ankle resistance with a unilateral soft exosuit increases plantarfexor efort during pushoff in unimpaired individuals | Participants didn’t meet criteria (healthy subjects) |
| 89. Thurston 2021 | Beyond orthoses: Using an exosuit to enhance the walking pattern of patients with unilateral Cerebral Palsy | Participants didn’t meet criteria (cerebral palsy) |
| 90. Siviy 2020 | Offline assistance optimization of a soft exosuit for augmenting ankle power of stroke survivors during walking | Outcome not of interest |
| 91. Lee 2019 | Long Shape Memory Alloy Tendon-  based Soft Robotic Actuators and Implementation as a Soft Gripper | Outcome not of interest |
| 92. Low 2018 | Design, characterisation and evaluation of a soft robotic sock device on healthy subjects for assisted ankle rehabilitation | Outcome not of interest |
| 93. Di Natali 2019 | Design and Evaluation of a Soft Assistive  Lower Limb Exoskeleton | Outcome not of interest |
| 94. Davila-Vilchis 2020 | Design Criteria of Soft Exogloves for Hand Rehabilitation-Assistance Tasks | Review article |
| 95. Butler 2020 | The ReStore Exosuit is a Safe Addition to Physical Therapy Treatment To Improve Walking Outcomes Post-Stroke | Same patient group with the included study (as Awad 2020) |
| 96. Jayaraman 2019 | Immediate Adaptations to Post-Stroke Walking Performance Using a Wearable Robotic Exoskeleton | Withdraw by journal |
